# Germline microRNA-based signatures predict toxicity and response to anti-CTLA-4 therapy

**DOI:** 10.1186/s12967-025-06842-3

**Published:** 2025-07-28

**Authors:** Joanne B. Weidhaas, Kristen M. McGreevy, Nicholas Marco, Nora Sundahl, Christopher R. Cabanski, Christine Spencer, Theresa LaVallee, Piet Ost, Donatello Telesca

**Affiliations:** 1 UCLA David Geffen School of Medicine, Los Angeles, CA USA; 2 Department of Biostatistics, UCLA, Los Angeles, CA USA; 3 Duke University, Durham, NC USA; 4 Department of Radiation Oncology, AZ Groeninge, Kortrijk, Belgium; 5 Parker Institute for Cancer Immunotherapy, San Francisco, CA USA; 6 Department of Human Structure and Repair, Ghent University, Ghent, Belgium

## Abstract

**Background:**

Germline microRNA-based variants (mirSNPs) have been shown to be predictive biomarkers of toxicity and tumor response across cancer treatments, including to anti-PD1/PDL1 immune checkpoint therapy. CTLA-4 inhibitors are another immune checkpoint inhibitor with known significant toxicity in the form of immune related adverse events (irAEs). The potential of mirSNPs to predict irAEs and/or response to anti-CTLA-4 therapy alone has not previously been reported and was the purpose of this investigation.

**Methods:**

We evaluated genetic signatures to predict toxicity and tumor response to anti-CTLA-4 treatment alone in melanoma patients using three separate cohorts. DNA was extracted from blood samples from 77 patients treated with anti-CTLA-4 therapy and analyzed using a custom panel of mirSNPs. We employed a combination of Elastic Net, Random Forest, and Boosted Tree models, incorporating germline mirSNPs, patient demographics, and treatment variables to predict toxicity in the form of irAEs or disease response. Additionally, we conducted a comparative analysis of gene ontology (GO) pathways to discern biological differences influenced by these genetic markers.

**Results:**

We developed two unique mirSNP signatures predicting toxicity or response to single agent anti-CTLA-4 treatment. These signatures both have excellent predictive accuracy with AUCs of 0.793 for toxicity and of 0.842 for response. The signatures do not overlap, nor is the toxicity signature similar to the toxicity signature for anti-PD1/L1 single agent therapy. Through GO analyses we found that both of these signatures have biological pathways involved in pri-miRNA transcriptional regulation, yet also have unique pathways that differentiate them.

**Conclusions:**

Our findings continue to support the utility of mirSNPs as predictive biomarkers of immune checkpoint therapy, for both toxicity and response. Further investigation in larger, diverse cohorts as well as to dual checkpoint inhibitor treatment is a planned next step to further their application.

**Supplementary Information:**

The online version contains supplementary material available at 10.1186/s12967-025-06842-3.

## Background

The advent of immune checkpoint inhibitors (ICIs) has redefined the therapeutic landscape for oncology treatment, using the body’s own immune system to detect and destroy cancer cells. Ipilimumab specifically targets the CTLA-4 pathway, a critical checkpoint that dampens immune activity [1], but its application as a monotherapy has been largely supplanted by combination regimens. Often given with anti-PD1/PDL1 therapy [2], together these checkpoint inhibitors consistently demonstrate enhanced response rates and survival benefits [3]. Objective response rates have climbed from 19% to a notable 58.3% with combination therapy, but this is offset by a stark increase in grade 3 or 4 toxicities, surging from 28 to 59% [4]. 

These toxicity events are a spectrum of autoimmune-like reactions across various organ systems induced by checkpoint inhibitors and are referred to as immune related adverse events (irAEs). irAEs in response to single agent anti-CTLA-4 therapy appear to be higher than to anti-PD1/PDL1 single agent therapy [5]. These irAEs pose substantial risks necessitating steroid intervention and therapy interruption, and can result in life long impacts on quality of life, including permanent endocrinopathies, adrenal insufficiency, cardiomyopathies, and other severe conditions that may shorten life [6]. 

In the absence of predictive biomarkers, the prevailing strategy to manage irAEs has been reactive rather than proactive, with a “watch and wait” approach post-treatment initiation. This reactive stance is particularly concerning as immune therapy gains traction across a broader array of tumor types, in younger patients, as well as in the adjuvant setting, thereby amplifying the urgency to identify individuals at higher risk for toxicities, even when they show a favorable response, to better inform balanced treatment plans.

Research continues to demonstrate that immune toxicity appears patient-specific, not tumor-specific, and can be predicted using germline DNA [7]. While protein-coding regions have had limited success in predicting toxicity to cancer treatment, in contrast, non-coding regions, like those coding for microRNAs, are gaining recognition for their potential as germline toxicity predictors. Progress in their application has been greatly enhanced by the identification of miRNA single nucleotide polymorphisms (mirSNPs) disrupting their circuitry [7–9]. Despite their significance, these mirSNPs remain underrepresented in routine SNP arrays and exome sequencing endeavors. mirSNPs have been shown by us and others to predict toxicity as well as response to anti-PD1/PDL-1 therapy [7, 9] but they have not previously been applied to single agent anti-CTLA-4 therapy, which is the purpose of this study.

## Methods

### Patients

This study incorporated melanoma patient cohorts treated with single agent anti-CTLA-4 therapy. Participants included ten patients with metastatic melanoma from Belgium treated on a trial which combined ipilimumab with stereotactic body radiation therapy (SBRT) (NCT02406183) [10]. Nine participants were from the Parker Institute, from the clinical trial NCT02731729, which compared ipilimumab alone versus a combination of ipilimumab and nivolumab in patients with Stage III-IV melanoma who had progressed or relapsed following anti-PD1 therapy [11]. Only those receiving ipilimumab monotherapy were included, all of whom had prior anti-PD1 treatments. Lastly, we obtained samples from a study at UCLA (PI: Dr. Ribas) of 58 patients with metastatic melanoma receiving anti-CTLA-4 therapy alone, without prior immune checkpoint inhibitors or concurrent radiation treatment.

irAEs were recorded by the treating physician and graded per American Society of Clinical Oncology guidelines as grades 1–4 or CTCAE V4.0 [12]. In this cohort irAEs were considered significant if they were recorded as grade *≥* 3.

Response was recorded by treating physicians using immune-Response Evaluation Criteria in Solid Tumors (iRECIST) or RECIST v1.1 criteria [13], using best overall response as follows: progressive disease (PD), stable disease (SD), partial response (PR), or complete response (CR). In this study, patients with SD, PR or CR were considered responders, versus those with PD who were considered non-responders. Clinically relevant variables, like anti-CTLA-4 cycle number, sex, age, and ethnicity, were collected. Patient demographics and outcomes are included in Table 1. *DNA Biomarker Panels*.

**Table 1:** Demographic information

		Toxicity		Disease Response	
	Overall	No Toxicity (Grade 0–2)	Toxicity (Grade 3+)	No Response (PD)	Response (CR, PR, SD)
	(*N* = 77)	(*N* = 61)	(*N* = 16)	(*N* = 51)	(*N* = 25)
Age					
Mean (SD)	59.9 (15.1)	58.1 (15.9)	66.9 (8.99)	59.0 (16.8)	62.5 (10.7)
Sex					
Female	21 (27%)	16 (26%)	5 (31%)	11 (22%)	10 (40%)
Male	56 (73%)	45 (74%)	11 (69%)	40 (78%)	15 (60%)
Ethnicity					
Hispanic	3 (3.9%)	3 (4.9%)	0 (0%)	3 (5.9%)	0 (0%)
White	34 (44%)	25 (41%)	9 (56%)	24 (47%)	10 (40%)
Missing	40 (52%)	33 (54%)	7 (44%)	24 (47%)	15 (60%)
Cycles CTLA4					
Mean (SD)	2.90 (1.51)	3.02 (1.54)	2.44 (1.31)	2.45 (1.38)	3.68 (1.35)
Toxicity Grade					
Mean (SD)	1.29 (1.22)	0.803 (0.853)	3.13 (0.34)	1.18 (1.24)	1.56 (1.16)
0	29 (38%)	29 (48%)	0 (0%)	22 (43%)	6 (24%)
1	15 (20%)	15 (25%)	0 (0%)	9 (18%)	6 (24%)
2	17 (22%)	17 (28%)	0 (0%)	11 (22%)	6 (24%)
3	14 (18%)	0 (0%)	14 (88%)	7 (14%)	7 (28%)
4	2 (2.6%)	0 (0%)	2 (13%)	2 (3.9%)	0 (0%)
Disease Progression					
Complete Response	4 (5.2%)	3 (4.9%)	1 (6.3%)	0 (0%)	4 (16%)
Partial Response	17(22%)	12 (20%)	5 (31%)	0 (0%)	17 (68%)
Stable Disease	4 (5.2%)	3 (4.9%)	1 (6.3%)	0 (0%)	4 (16%)
Progressive Disease	51 (66%)	42 (69%)	9 (56%)	51 (100%)	0 (0%)
Missing	1 (1.3%)	1 (1.6%)	0 (0%)	0 (0%)	0 (0%)

DNA was isolated from blood samples using Qiagen kits per protocol. The panel of mirSNPs were previously chosen based on their roles in disrupting DNA repair and immune-related genes [8]. All samples were processed and tested on an Agena MassArray platform.

### Pre-filtering SNPs

In order to limit the number of mirSNPs used to predict toxicity or response, we performed pre-filtering across the panel of 139 mirSNPs [8]. mirSNPs were included as potential covariates if they were marginally associated with our outcome of interest (either toxicity or response) via Fisher’s Exact Test [14] to analyze genotype distribution differences or Jonckheere-Terpstra Test [15] for ordered genetic effects at a liberal p-value threshold of 0.2. All p-values were calculated exactly via Monte Carlo simulations. The choice of the liberal p-value threshold is for reducing dimensionality, not for associative means. The 0.2 threshold (twice what would be considered barely significant) is expected to be robust across data and prediction scenarios.

### Model Building

We used germline mirSNPs, age, and sex to predict toxicity (grade ≥ 3) following ipilimumab treatment. We similarly used germline mirSNPs, age, sex, and cycles of ipilimumab treatment to predict disease response (CR, PR, SD versus PD) following ipilimumab treatment. We excluded cycles of ipilimumab from toxicity models as those who had toxicity stopped treatment; therefore, cycle of treatment was confounded with the toxicity outcome. Differently, cycle of treatment may be an important determinant of response and was therefore included in these models. We considered all toxicity regardless of type and classified them only by grade. We adopt a model-agnostic approach, by comparing several algorithms, e.g. Elastic Net (EN) [16], Random Forest (RF) [17], and Boosted Tree (BT) [18] to predict the outcomes of interest. Because machine learning algorithms (such as RF and BT) are sensitive to imbalances between groups, we up-sampled to balance the classes (ex: toxicity or not). In addition, we compare our genetic models to clinical only models. These models use the same clinical information: age and sex OR age, sex, and cycles for toxicity and response models, respectively.

Maximizing area under the curve (AUC) was used as the training metric for EN and RF models. The optimal lambda for EN models was chosen using 10-fold cross validation. For RF models, the number of trees varied between 50, 100, and 150 and the number of variables for each decision tree varied between 1 and 3. Unlike EN and RF models, BT models minimize deviance (log likelihood loss) when training the model. We used 0.6 bagging fraction with the Bernoulli distribution and varied depth between 1 and 3 and number of trees between 50, 100, and 150. By varying and comparing the number of trees, depth, and alpha (for EN), we used this as our hyperparameter tuning approach. Predicted values over 0.5 were classified as 1 and equal or under 0.5 as 0.

Nested leave-one-out cross validation (LOOCV) was used to evaluate model performance using sensitivity, specificity, positive predictive value (PPV), negative predictive value (NPV), F1 score, and AUC as metrics. To do this, we removed one observation at a time and built each model with the remaining observations. We used this model to predict the left-out observation’s toxicity or response and gathered all LOOCV predictions to calculate the metrics above. The model with the highest LOOCV AUC (between all EN, RF, and BT) was chosen and re-fit using all observations.

### Variable importance

Variable importance was calculated through a filtering approach, by dropping one variable and then refitting the model and calculating the AUC of the reduced model. The AUC of the reduced model was then compared to the AUC of the full model. A large decrease in AUC when removing a variable from the model would indicate that the variable is very important to discriminating and predicting whether or not a person will experience toxicity or response after the treatment [19].

### Association between toxicity and response

We examined whether the previous signature developed to predict toxicity to anti-PD1/PDL1 therapy was predictive of toxicity to anti-CTLA-4 therapy using a Fisher’s Exact test. Similarly, we examined if there is an association between toxicity and response to anti-CTLA-4 treatment.

### Comparative analysis of gene ontology (GO) pathways

We performed a stratified gene ontology (GO) analysis [20] to assess biological differences between mirSNPs involved in the toxicity and response signatures. mirSNPs were categorized into one of four distinct clusters: neither signature, both signatures, toxicity-only signature, and response-only signature. The mapIds function in R was used to annotate each mirSNP to its corresponding gene. Subsequent GO analysis was conducted, comparing gene clusters across groupings using the compareCluster and enrichGO functions in R. We applied an adjusted p-value cutoff of 0.05 using a universal genomic background. To account for our mirSNP pre-selection bias, we report pathways enriched in both, response, and toxicity signatures that were not enriched in the “neither” group. Further details on our GO analysis methods can be found in Supplemental File 1.

## Results

### Study patients

The study included 77 melanoma patients from three cohorts, with a larger proportion of males (*N* = 56) than females (*N* = 21) with the ages of the patients ranging from 23 to 89 with a mean age of 60 years old. Sixteen patients experienced grade *≥* 3 toxicity, with most experiencing gastrointestinal toxicities like diarrhea and colitis (Supplemental Table 1). Patients who experienced toxicity were about 9 years older on average compared to those who did not experience toxicity. As expected, patients who experience toxicity received fewer cycles of treatment (2.44) compared to those without toxicity (3.02), likely stemming from treatment discontinuation due to adverse events.

We had disease assessment data for 76/77 patients, and in total, there were 25 (33%) patients who responded (CR/PR/SD). Amongst responders, grade 0–3 toxicities were each observed in 6–7 patients. Basic demographics are detailed in Table 1.

### SNP filtering results

Twenty-seven out of 139 mirSNPs were marginally associated to toxicity via Fisher’s Exact or Jonckheere-Terpstra tests at p-value < 0.2 (Supplemental Table 2). Many SNPs appear to increase the proportion of toxicity, but SNPs in IL10RB and CD274 may be protective of toxicity. The proportion of toxicity in people who have 1 SNP is approximately half that of wildtype (29% vs. 19% IL10RB and 30% vs. 14% CD274), and the proportion of toxicity with 2 SNPs is 0% in both. Twenty-two out of 139 SNPs were marginally associated to response via Fisher’s Exact or Jonckheere-Terpstra tests at p-value < 0.2 (Supplemental Table 3).

### Models of toxicity and response

We evaluated model performance for predictive models of toxicity or response via nested leave-one-out cross-validation (LOOCV). Our toxicity (grade ≥ 3) model was an EN model with a LOOCV AUC of 0.793 (Table 2, Supplemental Fig. 1). This model is a large improvement over the best performing clinical only model, which achieved a LOOCV AUC of 0.652. Compared to this clinical model using just age and gender, mirSNPs improved the AUC by 0.14, and has balanced sensitivity and specificity (0.750, 0.836). The most important variables in the toxicity model were age and mirSNPs in TLR4 and TNNT2 (Table 3). People who are older have an increased risk of toxicity, and our model estimates every one year older someone is increases the odds of toxicity by about 2.23%.

**Table 2:** LOOCV performance metrics for toxicity and response prediction models

Model	Sensitivity	Specificity	PPV	NPV	F1	AUC
**Toxicity (Grade > = 3)**						
SNPs + Clinical	0.750	0.836	0.545	0.927	0.632	0.793
Clinical Only (age + gender)	0.500	0.803	0.400	0.860	0.444	0.652
**Response (CR**,** PR**,** SD)**						
SNPs + Clinical	0.840	0.843	0.724	0.915	0.778	0.842
Age, Sex, Ipi cycles only	0.720	0.608	0.474	0.816	0.571	0.664

**Table 3:** Most important model variables

Toxicity		Response	
Variable	Change in AUC	Variable	Change in AUC
Age	0.086	Cycles of CTLA4	0.080
TLR4	0.079	SPI1	0.080
TNNT2	0.054	FCGR2A	0.059
IL2RA	0.048	BMP2	0.050
ATM	0.046	IL10RB	0.030

Our response model (CR, PR, SD versus PD) was an Elastic Net model with a LOOCV AUC of 0.842 (Table 2, Supplemental Fig. 1). The best performing clinical model (using age, sex, and cycles of treatment) had a LOOCV AUC of 0.664, thus mirSNPs improved the AUC by 0.178 points. The response model also had high and balanced metrics, with almost identical sensitivity and specificity (0.840 and 0.843). Cycles of treatment and the SPI1 SNP were the most important variables for this model, each improving the AUC by 0.08 points. The proportion of response was 69% in people with 2 SNP copies compared to 24% and 28% in wildtype and heterozygous people (Supplemental Table 3). Neither age nor sex emerged as an important clinical variable in our response model.

### Signatures across immune checkpoint inhibitor types and for toxicity versus response

We assessed whether a previously developed signature for predicting toxicity to anti-PD1/PDL1 therapy would predict toxicity to anti-CTLA-4 therapy. Our analysis found no significant association between the predicted toxicity to anti-PD1/PDL1 therapy and the observed toxicity to anti-CTLA-4 therapy, with a Fisher p-value of 0.364 (Supplemental Table 4). This indicates that genetic predispositions conferring susceptibility to toxicity from anti-PD1/PDL1 therapy do not necessarily correlate with a heightened genetic risk for toxicity from anti-CTLA-4 treatment.

Next, we explored the association if any between toxicity and response to anti-CTLA-4 treatment. Again, our results indicated no significant link between experiencing toxicity and the treatment response (Fisher p-value = 0.372, Supplemental Table 4). This indicates that this signature for toxicity to anti-CTLA-4 therapy is not a predictor of treatment response to anti-CTLA-4 therapy.

### Anti-CTLA-4 toxicity and response GO pathways

**Fig. 1:**
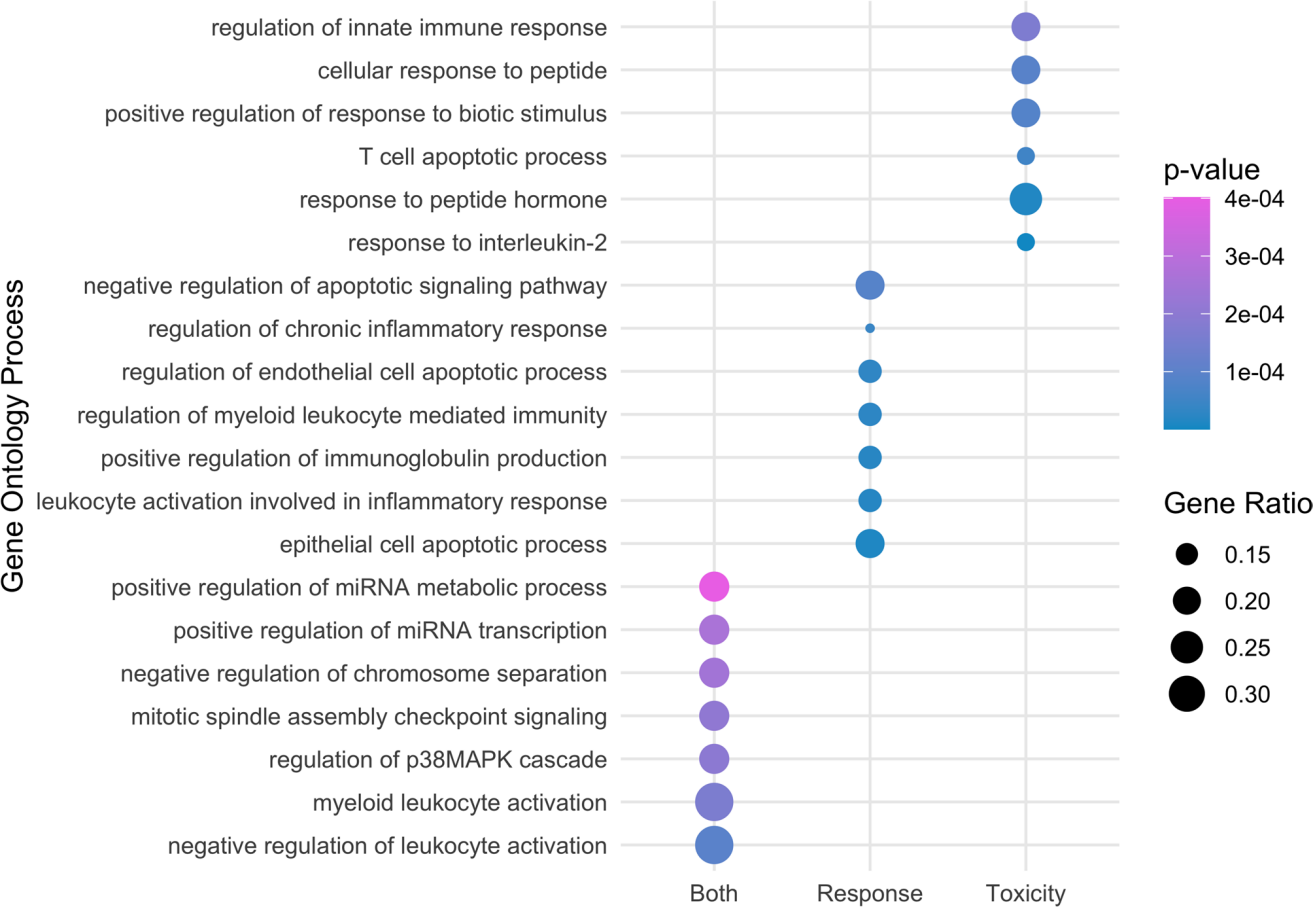
Top Gene Ontology Pathways enriched in Both, Toxicity, or Response Signatures for Anti-CTLA-4 Therapy

The GO analysis revealed 76 pathways enriched in both the toxicity and response signatures, 165 unique to the response signature, and 175 specific to the toxicity signature. This includes only pathways with at least 2 genes enriched compared to a universal genomic background and not enriched in the left-out panel mirSNPs at an adjusted p-value threshold of 0.05. Notably, pathways enriched in both signatures were predominantly involved in immune system regulation, cell cycle regulation, and miRNA regulation (Supplemental Table 5, Fig. 1). SPI1 gene is involved in many of the top GO processes.

The top GO terms found from the toxicity signature relate to cytokine response and cellular signaling and response. The top GO terms found from the rResponse signature relate to apoptosis and immune response regulation. Top GO results are presented in Supplemental Tables 5, and all significant GO pathways are provided in Supplemental Table 6.

## Discussion

In this study we evaluated the potential power of germline mirSNPs to predict toxicity and response to single agent anti-CTLA-4 therapy. We found that we were able to predict both treatment response and toxicity, and importantly, we found that these signatures differed from each other and from a previously identified germline signature of toxicity to single agent anti-PD1/PDL1 therapy. Our toxicity signature was predictive of immune related toxicity regardless of specific tissue-types of toxicity. We furthermore evaluated our biomarkers using a GO analysis, to develop insight into the potential pathways important in response and toxicity to anti-CTLA-4 checkpoint therapy.

In our GO analyses, biological pathways including immune system, cell cycle, and miRNA regulation played roles in both toxicity and response models for CTLA-4 treatment, yet divergent pathways suggest distinct genetic bases for favorable responses versus toxicity predispositions. GO analysis for toxicity highlighted the potential for external stimuli to be involved in immune checkpoint-inhibitor-induced toxicity, with interleukin signaling pointing towards a potential immunogenic or inflammatory pathway. Additionally, the regulation of response to biotic stimulus may illustrate cellular adaptations to biotic stress, relevant to immune reactions against cancer presence and therapeutic agents like anti-CTLA-4 antibodies. In contrast, top GO terms for response highlighted apoptotic roles and may suggest increased apoptosis in responders, which is part of the process that allows immune therapy to destroy tumor cells. This emphasizes anti-CTLA-4’s dual role in promoting cell death and modulating immune responses.

As far as the importance of individual mirSNPs, a mirSNP in TLR4 was identified as a central component of our genetic toxicity signature. TLR4 is a well-characterized pattern recognition receptor of the innate immune system, responsible for detecting pathogen-associated molecular patterns and triggering early inflammatory responses [21]. Importantly, TLR4 activation not only orchestrates innate immune defenses but also shapes subsequent adaptive immunity by regulating cytokine release and antigen-presenting cell function [22]. This dual role may underlie the association we observe between TLR4 genetic variation and immune-related adverse events (irAEs) in patients receiving immunotherapy for melanoma. The convergence of our TLR4 findings with enriched GO terms related to cytokine signaling, innate immune regulation, and cellular responses suggests that variation in innate immune sensors like TLR4 can modulate the risk of treatment toxicity, likely by amplifying or altering downstream inflammatory and adaptive immune mechanisms. These results emphasize the need to consider both innate and adaptive immune genetic variation in predicting and managing irAEs during cancer immunotherapy.

A mirSNP in the SPI1 gene emerged as the most important genetic predictor of response to ipilimumab treatment. This finding aligns with recent research identifying SPI1 as a prognostic biomarker for immunotherapy efficacy in clear cell renal cell carcinoma (ccRCC) [23]. The authors found that non-responders to immunotherapy against ccRCC were more likely to have higher SPI1 expression levels than responders. Additionally, Feng et al. demonstrated that high SPI1 expression was associated with increased infiltration of immune cells, particularly T cells and macrophages, which are crucial for an effective antitumor response [23]. This aligns with our observation that variations in SPI1 mirSNPs contribute to the variability in response. Our analysis indicates that the presence of SPI1 SNPs could be protective and increases the probability of a positive response to anti-CTLA-4 therapy in a dose-dependent manner. As mirSNPs affect gene expression or function, it is likely that this particular mirSNP influences the patient’s response to therapy in a similar manner.

A growing array of biomarkers, including tumor mutational burden (TMB) and PD-L1 expression, have been developed to predict ICI efficacy [24]. TMB, measured through tumor or circulating cell-free DNA (cfDNA), captures the somatic mutational landscape and is associated with beneficial response by increasing the potential for neoantigen presentation. However, in the process of calculating TMB, germline variants are purposefully excluded [25], and it is limited to exonic genomic regions [25], omitting potentially influential non-coding and regulatory regions of the genome. Recent work has shown that germline variants can influence TMB levels and the molecular phenotypes of tumors, referred to as germline variants influencing TMB (GVITMB) [26]. In our analysis here, we identified germline non-coding variants, including mirSNPs in DNA repair genes such as ATM and BRCA1, which directly align with research finding alternative, non tumor-tissue dependent biomarkers to predict TMB [27], supporting the concept that inherited genetic variation contributes to tumor biology and treatment response, by potentially shaping the molecular landscape in which somatic mutations arise.

PD-L1 expression is another FDA-approved biomarker for ICI efficacy [24]. PD-L1 expression has heterogeneity within tumors, changes expression over time and through treatment [28], and has technical limitations in detection [29]. Staining outcomes can be confounded by factors such as PD-L1 glycosylation [29], and the need for tumor tissue further limits accessibility. Moreover, PD-L1 expression is not always predictive of ICI benefit, as patients with low PD-L1 but high pathway engagement can still respond well to therapy [30]. Additional studies even point to genetic relationships of PD-L1 expression itself in *KRAS* mutations and mRNA regulation [31], further illustrating the interplay between inherited genetics and tumor-immune interactions.

Our germline-based approach adds a new and underexplored dimension to the biomarker landscape, complementing established somatic and tumor-derived biomarkers with stable, lifelong genetic information. In addition, and importantly, germline genetics can identify patients at increased risk of toxicity, which TMB and PD-L1 expression cannot. Our signatures capture components of established biology relating to ICI efficacy- including ATM, BRCA1, apoptosis, DNA repair, and immune cell pathways- while also highlighting the novel role of non-coding and miRNA regulatory variants in anti-CTLA-4 response and toxicity.

Our study has some limitations worth mentioning. First, our predictive signatures were developed using a relatively small cohort (*N* = 77), which may increase the risk of selection bias and limit the generalizability of our findings. While the sample size is modest, our study’s selective inclusion criteria likely contributed to the high AUC observed, as patients were drawn from defined oncological backgrounds and treated with single agent anti-CTLA-4 therapy. Second, there was heterogeneity in prior treatments for these patients, and all participants had melanoma, and thus it remains unclear whether these genetic signatures will generalize to other cancer types treated with anti-CTLA-4 therapy. Furthermore, although independent external validation of our findings is critical, we face the challenge that single agent anti-CTLA-4 therapy is no longer delivered to patients, and existing genomic data sets do not include the majority of the mirSNPs which we study. These are challenges that we plan to try to address with broader data sets of recently treated patients given combinations of anti-PD1 and anti-CTLA-4 therapy versus anti-PD1 therapy alone, where we can investigate if signatures of response and toxicity are similar to those found here with single agent anti-CTLA-4 therapy, as well as how our findings apply across cancer types in larger cohorts. These studies are ongoing, with promise for clinical utility of our biomarkers to help identify patients where one therapy, either anti-PD1/PDL1 alone, or combination anti-PD1 and anti-CTLA-4 therapy may be more efficacious, considering safety.

In summary, our research contributes a novel perspective to the understanding of immune checkpoint therapy, highlighting the role of mirSNPs in predicting both the therapeutic response and the risk of toxicity to anti-CTLA-4 therapy. By delineating the distinct genetic signatures and pathways associated with these outcomes, our work provides a foundation for future research aimed at optimizing treatment strategies and enhancing patient outcomes with personalized medicine.

## Electronic supplementary material

Below is the link to the electronic supplementary material.

Supplementary Material 1

Supplementary Material 2

Supplementary Material 3

Supplementary Material 4

Supplementary Material 5

Supplementary Material 6

Supplementary Material 7: Figure 1. Receiver Operator Characteristic Curves for **A**) Toxicity and **B**) Response Models

Supplementary Material 8

## Data Availability

Data are available upon reasonable request.
