# Supplementary figures and images for: Germline microRNA-based signatures predict toxicity and response to anti-CTLA-4 therapy
Source: J Transl Med. 2025 Jul 28;23:848. doi: 10.1186/s12967-025-06842-3 (PMC12306077; doi:10.1186/s12967-025-06842-3)

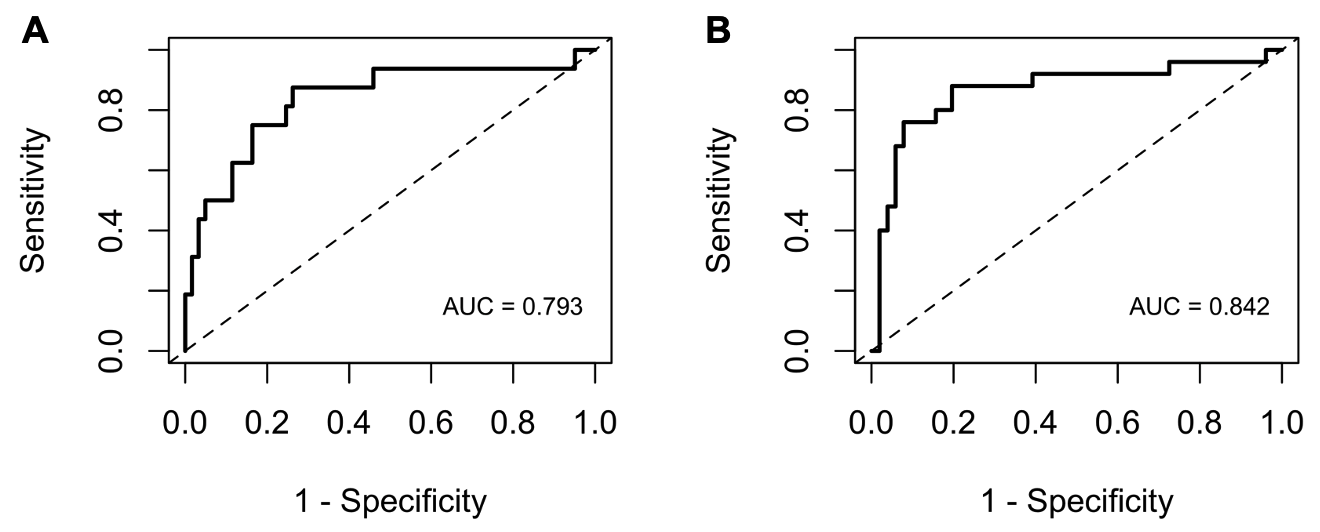

Supplement: Supplementary file 7 — Supplementary Material 7: Figure 1. Receiver Operator Characteristic Curves for A) Toxicity and B) Response Models [file 12967_2025_6842_MOESM7_ESM.png]
