# Supplementary material for: Germline microRNA-based signatures predict toxicity and response to anti-CTLA-4 therapy
Source: J Transl Med. 2025 Jul 28;23:848. doi: 10.1186/s12967-025-06842-3 (PMC12306077; doi:10.1186/s12967-025-06842-3)
Supplement: Supplementary file 8 — Supplementary Material 8 [file 12967_2025_6842_MOESM8_ESM.docx]

Supplemental File 1

In conducting our GO analysis to explore the biological underpinnings of mirSNPs associated with both toxicity and response signatures, we adopted a refined analytical strategy to address inherent biases and ensure the robustness of our findings. Recognizing the potential limitations introduced by the pre-selected nature of our mirSNP panel, which was inherently biased towards oncological relevance, we opted for a stratified GO analysis against a universal genomic background (as opposed to a background of the mirSNP panel). This approach was necessitated by several key considerations:

1. Pre-selection Bias: Our initial mirSNP panel was curated with an emphasis on oncology, predisposing it to a selection bias. Such a bias could skew the GO analysis, artificially inflating the biological significance of processes already known to be associated with cancer. By comparing against a universal genomic background, we mitigate this risk, enabling a more neutral evaluation of the mirSNPs' biological relevance.

2. Comprehensive Biological Context: A universal genomic background provides a broader context for evaluating the enrichment of specific GO terms. This comprehensive backdrop is crucial for detecting subtle, yet potentially significant, biological processes that may be overshadowed when using a more restricted reference set. It allows for the identification of unique biological themes that transcend the confines of the pre-selected SNP panel, offering novel insights into the mechanisms driving toxicity and response to immunotherapy.

3. Enhanced Specificity of Enrichment Analysis: By categorizing mirSNPs into distinct clusters (Neither Signature, Both Signatures, Toxicity-Only, and Response-Only) and conducting enrichment analyses that compare these clusters against a universal backdrop, we enhance the specificity of our findings. This methodology ensures that identified pathways are not only statistically significant but also biologically meaningful, reflecting genuine differences in the underlying mechanisms of toxicity and response.

4. Addressing the Challenge of Enrichment in a Pre-Enriched Set: Given the inherent enrichment of cancer-related processes within our initial mirSNP panel, identifying additional enrichment within this already biased group poses a methodological challenge. The use of a universal genomic background circumvents this issue, enabling the detection of enrichment that is not merely a reflection of the initial selection criteria but indicative of deeper biological significance.

By applying an adjusted p-value cutoff of 0.05 and reporting pathways enriched across the specified mirSNP clusters while explicitly contrasting these with the "Neither" group, we ensure that our findings are not only statistically robust but also of clear biological relevance. This approach provides a solid foundation for understanding the complex interplay between genetic variants and the immune response in the context of cancer therapy, paving the way for the identification of novel biomarkers and therapeutic targets.
